# Supplementary material for: Correlation of NTRK1 Downregulation with Low Levels of Tumor-Infiltrating Immune Cells and Poor Prognosis of Prostate Cancer Revealed by Gene Network Analysis
Source: Genes (Basel). 2022 May 8;13(5):840. doi: 10.3390/genes13050840 (PMC9140438; doi:10.3390/genes13050840)
Supplement: Supplementary file 1 [file genes-13-00840-s001.zip › Supplementary Figures.pdf]

Supplementary Figures

Correlation of NTRK1 Downregulation with Low Levels of Tumor-Infiltrating Immune Cells and Poor Prognosis of Prostate Cancer Revealed by Gene Network Analysis

Arash Bagherabadi [1], Amirreza Hooshmand [2], Nooshin Shekari [3], Prithvi Singh [4], Samaneh Zolghadri [2,*,†], Agata Stanek [5,*,†] and Ravins Dohare [4,†]

[1] Department of Biology, Faculty of Sciences, University of Mohaghegh Ardabili, Ardabil 56199-11367, Iran; a.bagherabadi@student.uma.ac.ir
[2] Department of Biology, Jahrom Branch, Islamic Azad University, Jahrom 7414785318, Iran; amirrezahoushmand66@gmail.com
[3] Department of Biology, Faculty of Sciences, Shahid Chamran University of Ahvaz, Ahvaz 6135783151, Iran; shekarinooshin51@gmail.com
[4] Centre for Interdisciplinary Research in Basic Sciences, Jamia Millia Islamia, New Delhi 110025, India; prithvi.mastermind@gmail.com (P.S.); ravinsdohare@gmail.com (R.D.)
[5] Department and Clinic of Internal Medicine, Angiology and Physical Medicine, Faculty of Medical Sci-ences in Zabrze, Medical University of Silesia, Batorego 15 St., 41-902 Bytom, Poland
* Correspondence: z.jahromi@ut.ac.ir (S.Z.); astanek@tlen.pl (A.S.); Tel.: +48-604-119-099 (S.Z.); +98-715-437-200 (A.S.)
† These authors have contributed equally to this work.

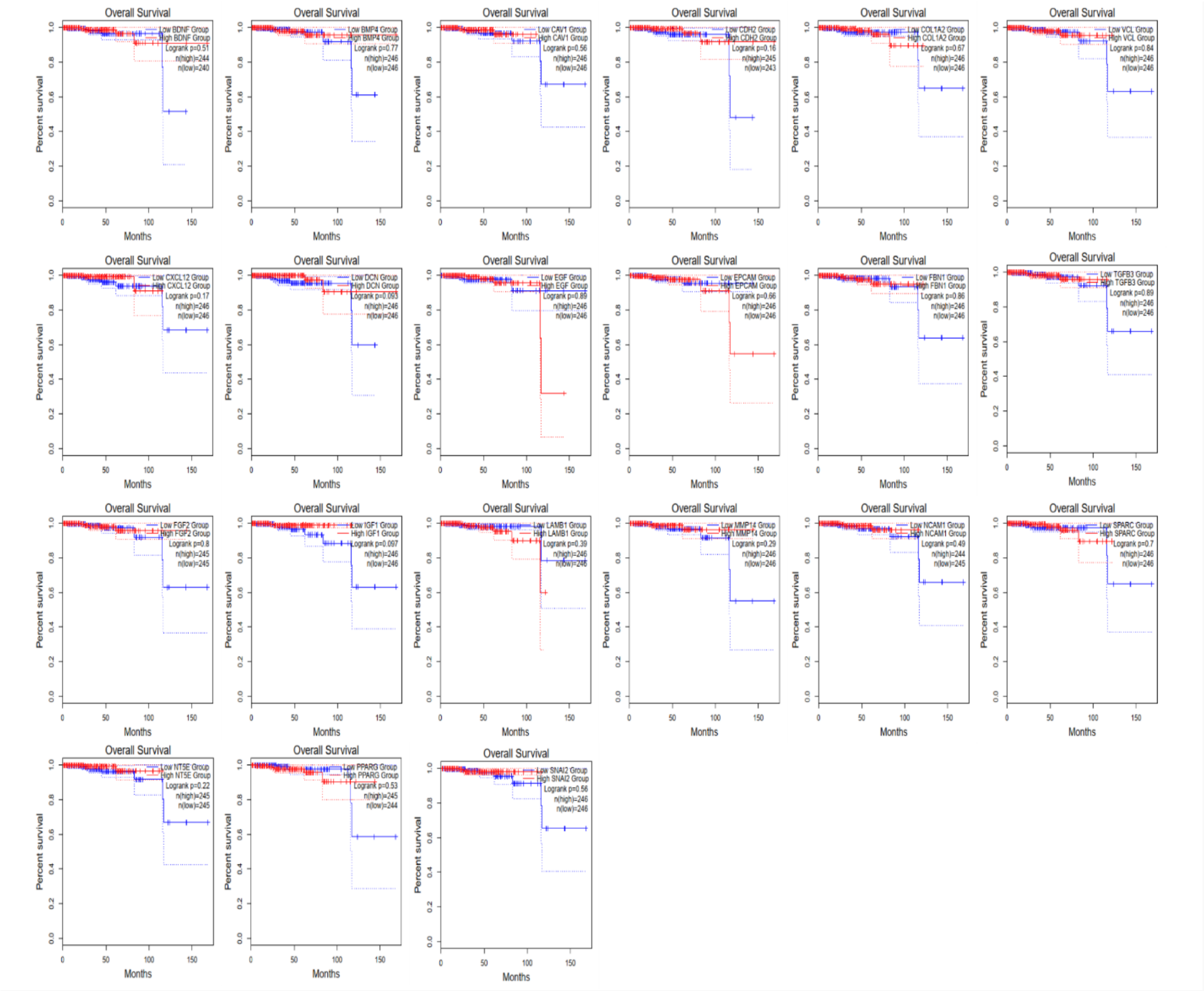

Figure S1. OS analysis of PCa HUBs based on the GEPIA database determined by the KM curve. The expression of other 21 HUBs was not shown to significantly impact the prognosis of PCa in using KM estimates (their Log-rank $p \geq 0.05$). HUBs, hub genes; PCa, prostate cancer; KM, kaplan-meier.

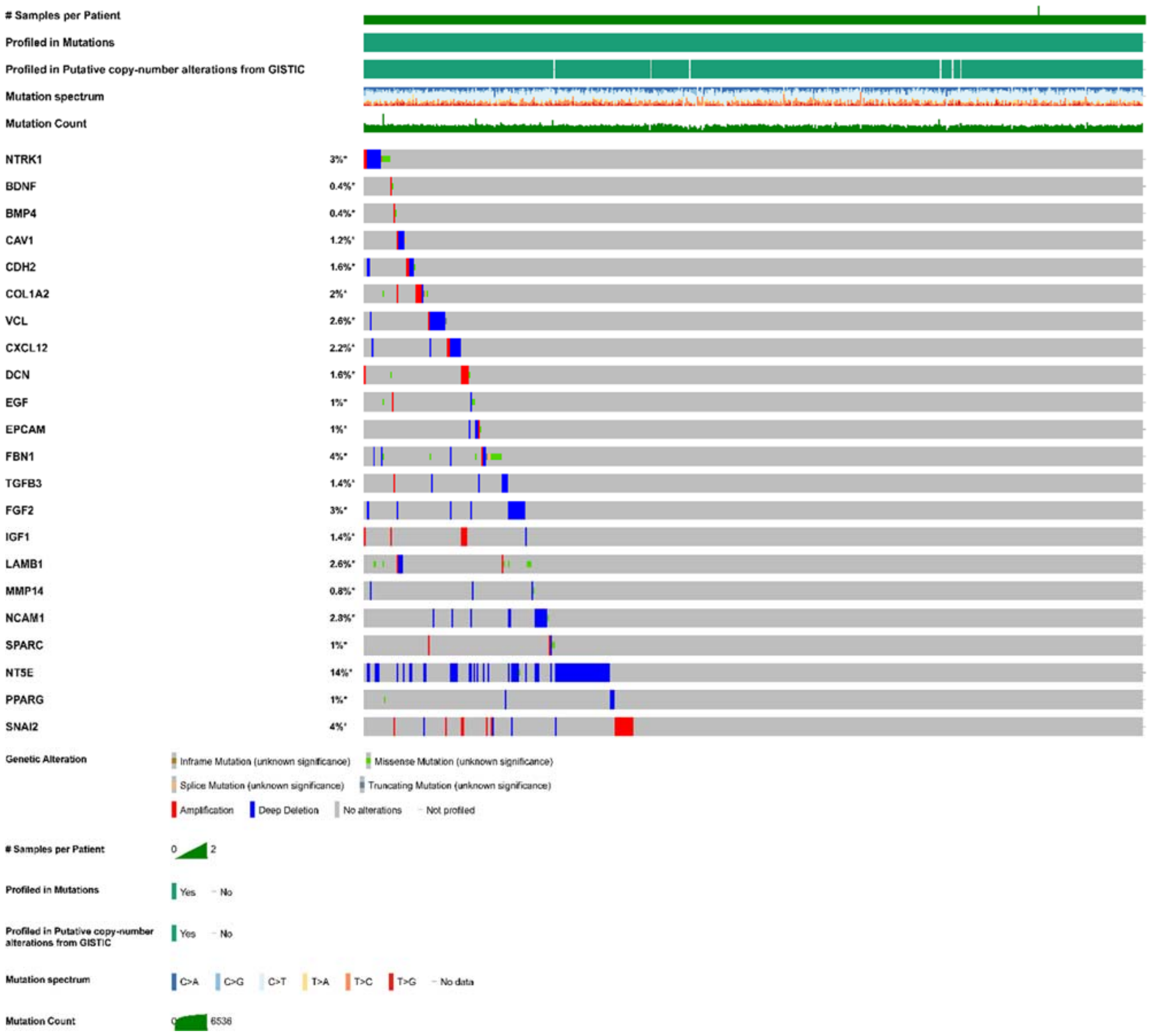

Figure S2. OncoPrint summarizes genomic alterations in 22 PCa HUBs across the TCGA-PRAD cohort comprising 501 patient samples. The bottom row represents frequency of genomic alterations in these HUBs with green, orange, grey, red, blue, and golden bars signifying missense, splice, truncating, amplification, deep deletion, and inframe mutations, respectively. First, second, third, fourth, and fifth rows depict the clinical annotation bars such as samples per patient, profiled in mutations, and putative copy-number alterations from GISTIC, mutation spectrum, and mutation count.
